# Biomarker-guided antibiotic therapy in adult critically ill patients: a critical review

**DOI:** 10.1186/2110-5820-2-32

**Published:** 2012-07-23

**Authors:** Pedro Póvoa, Jorge I F Salluh

**Affiliations:** 1 Polyvalent Intensive Care Unit, Hospital de São Francisco Xavier, Centro Hospitalar de Lisboa Ocidental, Estrada do Forte do Alto do Duque, Lisbon 1449-005, Portugal; 2 CEDOC, Faculty of Medical Sciences, New University of Lisbon, Lisbon Portugal; 3 D’Or Institute for Research and Education, Rio de Janeiro, Brazil; 4 Postgraduation Program, Instituto Nacional de Câncer, Rio de Janeiro, Brazil

**Keywords:** Antibiotic stewardship, Biomarkers, C-reactive protein, Infection, Procalcitonin, Sepsis

## Abstract

Biomarkers of infection, namely C-reactive protein and procalcitonin (PCT), are potentially useful in the diagnosis of infection as well as in the assessment of its response to antibiotic therapy. C-reactive protein variations overtime appears to have a good performance for the diagnosis of infection. Procalcitonin shows a better correlation with clinical severity. In addition, to overcome the worldwide problem of antibiotic overuse as well as misuse, biomarker guidance of antibiotic stewardship represents a promising new approach. In several randomized, controlled trials, including adult critically ill patients, PCT guidance was repeatedly associated with a decrease in the duration of antibiotic therapy. However, these trials present several limitations, namely high rate of patients’ exclusion, high rate of algorithm overruling, long duration of antibiotic therapy in the control group, disregard the effect of renal failure on PCT level, and above all a possible higher mortality and higher late organ failure in the PCT arm. In addition, some infections (e.g., endocarditis) as well as frequent nosocomial bacteria (e.g., *Pseudomonas aeruginosa*) are not suitable to be assessed by PCT algorithms. Therefore, the true value of PCT-guided algorithm of antibiotic stewardship in assisting the clinical decision-making process at the bedside remains uncertain. Future studies should take into account the issues identified in the present review.

## Review

### Introduction

During the past three decades, the incidence of sepsis has been consistently rising, surpassing that of cardiac failure and has an annual mortality rate above acute myocardial infarction [1,2]. A recent report from the Healthcare Cost and Utilization Project found that hospital costs increased almost 25 % (inflation-adjusted) from 2001 to 2007 [3]. Among the top ten conditions with the most rapidly increasing hospital costs, three were infections, with “blood infection (septicemia)” showing the largest growth in cumulative costs (174.1 %) and the highest aggregate costs (12.3 billion USD in 2007).

Consequently sepsis is a major public health issue that can affect all population, including healthy people, although there is a predominance in elderly patients with multiple or severe comorbidities. However, during the past 15 years, several reports showed a sustained and continuous improvement in mortality of severe sepsis and septic shock, although it remains exceedingly high, ranging between 30-50 % [4-6]. Yet, this improvement in outcome cannot be attributed to the introduction of a new drug or treatment but to an improvement of the process-of-care with more rapid diagnosis and treatment [6,7]. Even though a delay in the institution of antibiotic therapy is markedly associated with a worse prognosis, antibiotic overuse should be avoided and interventions to reduce the duration of antibiotic therapy were associated with lower mortality and length of stay as well as a decrease in the prevalence of multidrug-resistant microorganisms [8,9].

With the goal of shortening the duration of antibiotic therapy, the efficacy of procalcitonin (PCT)-guided antibiotic stewardship algorithms have been tested in different clinical settings [10]. As a result, the discussion of the available strategies to decrease the duration of antibiotic therapy, with and without biomarkers, in particular in the critical care setting, seems timely and relevant.

### Can biomarkers be used to guide antibiotic therapy in severe sepsis?

The diagnosis of infection is not straightforward, because there is no “gold standard” test [11]. Consequently, antibiotics are frequently prescribed without a definite diagnosis, because a delay in treatment is associated with decreased survival [12]. Even more difficult than diagnosis, is monitoring of infection response to antibiotics [13]. Currently, the assessment of response relies on the resolution of the same criteria used in the diagnosis. Because the inflammatory cascade plays a central role in host-pathogen interaction and in infection control mechanisms, these mediators have been assessed as surrogate markers of infection, both in diagnosis and in monitoring response [11]. Unfortunately the “ideal” biomarker has not yet been discovered (Table 1).

**Table 1:** **Characteristics of the ideal biomarker of infection**[13,14]

1 Easy to use and interpret	
2 Objective	
3 Rapidly available	
4 Reproducible	
5 Good sensitivity and good specificity	
6 Dynamic – rapid increases and decreases	
7 Level not dependent of the underlying pathology and not modified by any treatment or intervention unless interventions related to the source control and/or antibiotic therapy	
8 Continuous and not a discrete variable	
9 Correlation with clinical severity and mortality	
10 Prolonged and successive infections without “exhaustion” or “fatigue”	
11 Inexpensive	
12 Easily available	

### Do we need biomarkers to guide/reduce antibiotic therapy in severe sepsis, namely in VAP?

Two landmark studies were published that evaluated the impact of shorter duration of antibiotic therapy in ventilator-associated pneumonia (VAP) [15,16]. The PneumA trial [15] was a prospective, randomized, controlled trial (RCT) in 51 French intensive care units (ICU) designed to assess whether 8 days was as effective as 15 days of adequate antibiotic therapy in microbiologically documented late-onset VAP (N = 402). The authors showed that an 8-day course of antibiotics was as effective as a 15-day treatment (all-cause mortality: 18.8 % vs. 17.2 %).

The second study was a single-center, prospective RCT designed to evaluate the effectiveness and safety of a discontinuation policy on the duration of antibiotic therapy of VAP (N = 290) [16]. The authors showed that an active discontinuation policy could safely decrease the duration of antibiotic therapy to 6 days (*p* = 0.001). Both groups presented similar hospital mortality and ICU and hospital length of stay (LOS) (*p* = 0.357, *p* = 0.798, *p* = 0.865, respectively). These findings suggest that shorter courses of antibiotic therapy, 6–8 days, in VAP can be safely achieved without the use of biomarkers.

### C-reactive protein and procalcitonin biology

Among all biomarkers of infection those more frequently studied are C-reactive protein (CRP) and PCT [11]. Before using these biomarkers in clinical practice, it is essential to know its biology, strengths, and limitations.

Plasma CRP, like all other acute phase proteins, is exclusively synthesized in the liver in response to interleukin 6 [17,18]. Four to 6 hours after an inflammatory insult, CRP secretion begins. Its concentration doubles every 8 hours and peaks at 36–50 hours [19-21]. With the elimination or removal of the primary inflammatory stimulus, CRP falls rapidly with a first-order kinetics pattern of elimination with a half-life of 19 hours [22]. C-reactive protein concentration rises whenever an inflammatory process is present and its serum concentration depends only on the intensity of the stimulus and on the rate of synthesis [19,21,22]. C-reactive protein level is independent of the underlying disease and is not modified by any therapy or intervention such as renal replacement therapy (RRT) [23], systemic steroids [24], or neutropenia [25].

The pathophysiological role of PCT in sepsis is not fully understood [10]. After an inflammatory stimulus, PCT is detectable as soon as 3-4 hours, peaking at 14-24 hours [26-29]. After removal of the inflammatory stimulus, PCT half-life ranged from 22-35 hours [29]. There are several well-recognized limitations to the use of PCT as a surrogate marker of infection. It has been shown that in septic cancer patients with leukopenia PCT concentrations were lower [30]. Besides renal function is a major determinant of PCT levels [10,31,32], and in addition, PCT is markedly cleared by different techniques of RRT [23,33].

### Assays to measure C-reactive protein and procalcitonin

The characteristics of the assays used to measure the marker are a fundamental aspect when dealing with biomarkers [34]. The available immunoassays of CRP measurement are reliable, stable, and highly reproducible [35]. Besides they are inexpensive (approximately 4€ in Europe), rapid (15–30 minutes), and with a limit of detection of 0.3-5 mg/L [36]. This limit of detection of CRP is acceptable to its utilization as a biomarker in diagnosis and in monitoring response to antibiotics [21].

To be useful at the bedside, the PCT assay should have a high sensitivity [10]. Only the immunoassay, based on a time-resolved amplified cryptate emission (TRACE) technology (Kryptor PCT assay, Brahms), has a reasonable limit of detection: 0.06 ng/mL [37]. The assay time is rapid (19 min) but still quite expensive (25-30€ in Europe) [10]. All the other less sensitive assays, namely the semiquantitative immunochromatographic method (PCT-Q, Brahms) and the luminescence immunoassay (PCT LIA, Brahms), should be used with caution and never for antibiotic stewardship [10,38,39].

### What is the PCT algorithm for stewardship of antibiotic therapy?

**Figure 1:**
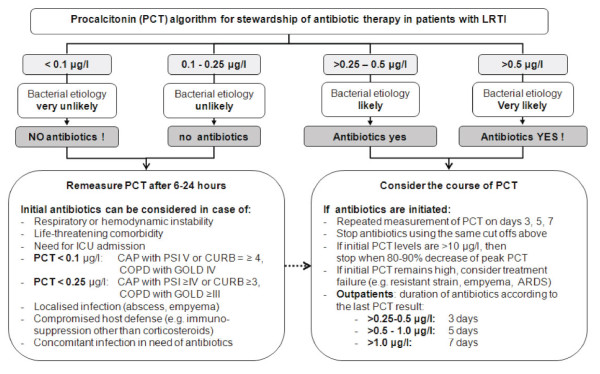
**Procalcitonin algorithm for stewardship of antibiotic therapy; adapted from [**[10]**]**.

The proposed algorithms on antibiotic stewardship are based on different PCT cutoff ranges. These cutoffs were derived from several well-conducted, prospective, observational studies and were validated in different RCT [40]. The PCT algorithm for primary care and emergency departments can be summarized as follows: antibiotics were more or less discouraged (<0.1 ng/mL or 0.1–0.25 ng/mL) or encouraged (>0.25–0.5 ng/mL or >0.5 ng/mL) [10]. According to these PCT cutoff ranges, bacterial etiology was considered *very unlikely, unlikely, likely*, and *very likely*, respectively [10]. To prevent not treating an infection, some overruling criteria were included in the algorithm (Figure 1) [10].

For patients in the ICU, the algorithm for antibiotic stewardship was similar but with somewhat higher PCT cutoff ranges [40]. Likewise, if antibiotics were withheld the algorithm recommends a clinical reevaluation and subsequent PCT measurement after 6-24 hours. In the case of antibiotic therapy, PCT should be monitored daily. Antibiotics should be discontinued when PCT decreases >80 % of the initial level or if an absolute PCT value <0.5 ng/mL is reached. Again, if PCT levels remain elevated, treatment failure, potential infectious complications or superinfection should be considered [40].

### Studies of PCT-guided algorithms of antibiotic stewardship in adult critically ill patients

#### Characteristics of selected RCT

A total of 7 RCT dealing with PCT-guided antibiotic stewardship in adult critically ill patients were identified for further discussion [41-47]. They were published between 2007 and 2011 (Table 2). A total of 2,190 adult critically ill patients were included in the above-mentioned RCTs (Table 2). From the included patients, 1,331 (60.8 %) presented nosocomial infections and the remaining 859 were community-acquired. Pneumonia was the most frequent infection in four studies, whereas peritonitis was more frequent in the other three (n = 33 [43], n = 19 [44], and n = 59 [46]).

**Table 2:** Principal characteristics of the randomized controlled trials assessing the role of PCT-guided antibiotic stewardship in adult critically ill patients

	**Trial**	**Sample size**	**Rate of exclusion (%)**	**Infections Community/nosocomial**	**Pneumonia**	**PCT assay**	**Minimum duration AB therapy**	**Decision to start antibiotics (no AB), PCT/control**	**Duration of antibiotic therapy, PCT/control, days**	**Overruling PCT algorithm (%)**	**LOS ICU, PCT/control, days**	**Superinfection, PCT/control, N(%)**	**Relapse, PCT/control, N(%)**	**Mortality 28d, PCT/control, N(%)**	**Mortality 60d, PCT/control, N(%)**
Svoboda, 2007 [43]		72	381 (84)	0/72	NA	PCT-Q					16.1 ± 6.9/19.4 ± 8.9			10/38 (26 %)/13/34 (38 %)	
Nobre, 2008 [41]	ProSEP	79	203 (72)	53/26	52	TRACE			8 (4–27)/14 (6–39)	19	4 (1–21)/7 (1–91)	7/31 (22.5 %)/11/37 (29.7 %)	1/39 (2.6 %)/1/40 (2.5 %)	8/39 (20.5 %)/8/40 (20 %)	
Schroeder, 2009 [44]		27	98 (78)	0/27	8	PCT LIA	yes		6.6 ± 1.1/8.3 ± 0.7		16.4 ± 8.3/16.7 ± 5.6			3/14 (21.4 %)/3/13 (23.1 %)	
Stolz, 2009 [45]	ProVAP	101	63 (38)	0/101	101	TRACE	yes		10 (6–16)/15 (10–23)	16	13 (7–21)/13.5 (8–22.2)	7/51 (13.7 %)/6/50 (12 %)		8/51 (16 %)/12/50 (24 %)	
Hochreiter, 2009 [46]	ProSICU	110	285 (72)	0/110	43	PCT LIA			5.9 ± 1.7/7.9 ± 0.5		15.5 ± 12.5/17.7 ± 10.1			15/57 (26.3 %)/14/53 (26.4 %)	
Bouadma, 2010 [42]	PRORATA	601	685 (52)	326/275	394	TRACE	yes	4 (1.7 %)/15 (4.8 %)	10.3 ± 7.7/13.3 ± 7.6	53	15.9 ± 16.1/14.4 ± 14.1	106/307 (34.5 %)/97/314 (30.9 %)	20/307 (6.5 %)/16/314 (5.1 %)	65/307 (21.2 %)/64/314 (20.4 %)	92/307 (30 %)/82/314 (26.1 %)
Jensen, 2011 [47]	PASS	1200	3 (0.3)	480/720	666	TRACE			6 (3–11)/4 (3–10)	17.9	6 (3–12)/5 (3–11)			190/604 (31.5 %)/191/596 (32 %)	231/604 (38.2 %)/220/596 (36.9 %)

Results are expressed as mean ± standard deviation or median (interquartile range) unless otherwise indicated.

AB = antibiotics; LOS = length of stay; PCT = procalcitonin; PCT LIA = luminescence immunoassay; PCT-Q = semiquantitative immunochromatographic method; TRACE = time-resolved amplified cryptate emission.

#### Reasons for exclusion of screened patients from the studies

The clinical impact of an intervention is somehow dependent on its applicability. In the seven selected RCT, the rate of exclusion ranges from 0.3 % [47] to 84 % [43]. However, with the exception of the larger RCT that presented the lower exclusion rate [47], in all the others the rates of exclusion were exceedingly high, 84 % [43], 72 % [41], 78 % [44], 38 % [45], 72 % [46], and 52 % [42].

It also is necessary to examine the criteria for exclusion. In three studies the description was very poor [43,44,46]. In the other RCT, it is clear that common ICU infections are excluded [41,42,45], namely caused by *Pseudomonas aeruginosaAcinetobacter baumannii*, as well as frequent clinical situations, namely endocarditis or infections that require long-course antibiotics and immunosuppressed patients.

#### PCT measurement methodology

The quality of the studies also is largely dependent of the methodology used to measure PCT plasma concentrations. One RCT used a semiquantitative immunochromatographic method [43], and two the less sensitive luminescence immunoassay [44,46], which are currently not recommended for antibiotic stewardship [10]. The ultrasensitive methodology was used in the remaining four trials [41,42,45,47].

#### PCT-guided algorithms of antibiotic stewardship

Each of the RCT describes a specific PCT-guided algorithm of antibiotic stewardship; however, as a whole, the main frame was quite similar to that previously described. In four RCT, the algorithm was designed to assist in the decision to stop antibiotics [41,44-46], in two to assist in the decision to start and to stop antibiotics [42,43], and finally to start or escalate antibiotics [47]. In two studies, the PCT guidance also impacted the management of patients because concomitant interventions were performed [43,47].

#### The standard of care

In the seven RCT, the control group was managed according to the standard of care. This is vague, because there is substantial difference between usual care, the standard of care, and the best care [48]. According to the above-mentioned VAP studies, the course of antibiotics could be safely reduced to 6–8 days [8,15,16].

Therefore, it would be important to know whether patients in the control group have a minimum duration of antibiotic therapy. This was the case in three RCT [42,44,45]. In one study, the antibiotic duration was calculated according to the underlying infectious pathology [44]. In other trial, dealing only with VAP, the minimum duration of antibiotic therapy in the control group was 15 days [45]. Finally, in the last trial, the recommendations were for community-acquired pneumonia 7–10 days with a longer duration, 14 days, if pneumonia was caused by *Legionella pneumophilaMycoplasma pneumoniae*, or *Chlamydia pneumoniae*, and for VAP 8–15 days [42].

This raises two issues. First, several years after the publication of RCTs showing that 6–8 days of antibiotic therapy in VAP is safe [15,16], it may not be acceptable to have control groups treated for so long. Second, the attending physician was not allowed to use the clinical and laboratory course of the patient as a stopping rule.

#### Impact of PCT-guided algorithm in the decision to start antibiotics

In two trials, the PCT-guided algorithm was designed to assist in the decision to start antibiotics [42,43]. In one study, all patients received antibiotics at inclusion; however, the criteria to start were not very clear and in addition the use semiquantitative method of PCT measurement precluded further comment [43]. In the PRORATA trial at inclusion, paradoxically more patients (N = 15) in the control group did not receive antibiotics, because the attending physician considered the patient not infected, than in the PCT-guided group (N = 4) [42].

#### Impact of PCT-guided algorithm in the decision to stop antibiotics

In five RCT, the PCT-guided algorithm was designed to assist in the decision to stop antibiotics [41,42,44-46]. All were able to decrease the duration of antibiotic therapy in ICU patients as well as the antibiotic-free days, 28 days after inclusion.

The duration, in days, of antibiotic therapy of the first episode of infection was shorter in the PCT-guided arm in all studies, 6 (3–34) vs. 9.5 (2–33) (p = 0.15) [41], 6.6 ± 1.1 vs. 8.3 ± 0.7 (p < 0.001) [44], 3 (0–8) vs. 5 (1–9.5) (*p* = not shown) [45], 5.9 ± 1.7 vs. 7.9 ± 0.5 (p < 0.001) [46] and 6.1 ± 6.0 vs. 9.9 ± 7.1 (*p* < 0.0001) [42]. Similarly, the total duration, in days, of antibiotic therapy was shorter in the PCT-guided group.

Consequently, the exposure to antibiotics, expressed by the antibiotic-free days, was lower in patients from the PCT arm [41,42,45].

#### Impact of PCT-guided algorithm in the decision to start and/or escalate antibiotics

Recently, the largest, multicenter trial to assess a PCT-guided algorithm was published: the PASS trial [47]. Its design was based on the finding of the so-called “alert PCT” [49]. It consists of PCT > 1.0 ng/mL or not decreasing <10 %/d to indentify infected patients at risk of complications or treatment failure. In this observational study, the presence of an “alert PCT” was significantly associated with 90-day all-cause mortality [49]. The interventions in the “alert PCT” days consisted on cultures, radiology, and empiric antibiotic therapy (prescription or broaden spectrum antibiotic according to an algorithm) with the goal to reduce mortality.

The use of large-spectrum antibiotics was substantially increased in the PCT arm with a shorter time to start (*p* < 0.0001) and a longer exposure (*p* < 0.001) [47]. However, the time until appropriate antibiotic therapy was similar between groups with the exception of bloodstream infections, shorter in the PCT-guided group [47]. Total duration of antibiotic therapy was longer in the PCT-guided arm: (median) 6 [range, 3-11] vs. 4 days [range, 3-10] (*p* < 0.05).

#### Compliance with PCT-guided algorithm

In addition to the extent of excluded patients, the degree of algorithm overruling is an indirect measure of its clinical applicability as well as of its true impact in the clinical decision making process. In the PRORATA trial [42], at inclusion 89 patients had a PCT <0.5 μg/L and according to the algorithm antibiotics were “discouraged” or “strongly discouraged.” However, in 73 % of patients (n = 65) the attending physician overruled this recommendation, because they considered the patient “infected” despite a low PCT level. This represents a 21 % of algorithm overruling at inclusion (65/307).

In three RCT that assessed the impact of PCT-guided algorithm in the decision to stop antibiotics, it was possible to assess the rate of overruling [41,42,45]. In two studies, the rate of overruling of stopping rules were 19 % (6/31) [41] and 16 % (8/51) [45]. In both cases, the attending physician prolonged antibiotic therapy despite a low PCT level. In the PRORATA trial [42], the reasons for overruling were: n = 39 patients, antibiotics were stopped despite a PCT > 0.5 μg/L, because infection was considered clinically cured; n = 79 patients, antibiotic therapy was prolonged despite a PCT < 0.5 μg/L, because patients were clinically unstable. Overall, the rate of PCT-guided algorithm was 53 % [42].

Finally, in the PASS trial [47], 56 of 312 (17.9 %) patients with baseline “alert PCT" did not receive antimicrobials. Unfortunately, none of the studies assessed specifically the outcomes of the patients with algorithm overruling.

#### Length of ICU stay and organ failure

All studies provided data concerning the LOS (in days) in the ICU that was very similar in the PCT and control groups: 16.1 ± 6.9 vs. 19.4 ± 8.9 [43], 4 (1–21) vs. 7 (1–91) [41], 16.4 ± 8.3 vs. 16.7 ± 5.6 [44], 13 (7–21) vs. 13.5 (8–22.2) [45], 15.5 ± 12.5 vs. 17.7 ± 10.1 [46], 15.9 ± 16.1 vs. 14.4 ± 14.1 [42], and 6 [3-12] vs. 5 [3-11][47].

An objective assessment of organ failures is provided in only two trials [42,47]. Baseline organ failures were similar in both groups. However, in the PRORATA trial, the SOFA score was higher in the PCT group at day 28 (*p* = 0.037) [42]. In the PASS study, the number of days on mechanical ventilation was significantly higher in the PCT-guided group: 3,569 days (65.5 %) vs. 2,861 days (60.7 %; *p* < 0 .001) [47].

#### Rates of superinfections and relapses

Three studies provide the rate of superinfections. One was lower in PCT-guided arm: 7/31 (22.5 %) vs. 11/37 (29.7 %) [41]. The other two were higher in the PCT group: 7/51 (13.7 %) vs. 6/50 (12 %) [45] and 106/307 (34.5 %) vs. 97/314 (30.9 %) [42].

Two studies provided data on the rates of infection relapse that were similar in one study: 1/39 (2.6 %) vs. 1/40 (2.5 %) [41]; the second was slightly higher in the PCT arm: 20/307 (6.5 %) vs. 16/314 (5.1 %) [45].

Finally, two studies monitored the emergence of multidrug resistance bacteria with very different overall rates. In the PRORATA study [42], the rate of multidrug resistance bacteria in the PCT arm was 17.9 % vs. 16.6 % (*p* = 0.67), whereas the PASS was significantly higher in the control group: 2.4 % vs. 3.1 % (*p* = 0.01) [47].

#### Mortality

All studies provided 28-day all-cause mortality rates that were comparable. A closer look showed that in four trials, the 28-day mortality was very similar: 8/39) (20.5 %) vs. 8/40 (20 %) [41]; 3/14 (21.4 %) vs. 3/13 (23.1 %) [44]; 15/57 (26.3 %) vs. 14/53 (26.4 %) [46]; 65/307 (21.2 %) vs. 64/314 (20.4 %) (*p* = NS) [42]; and 190/604 (31.5 %) vs. 191/596 (32 %) [47]. In two trials, the mortality in the control group was higher, although not significantly: 10/38 (26 %) vs. 13/34 (38 %) (*p* = 0.28) [43] and 8/51 (16 %) vs. 12/50 (24 %) (*p* = 0.327) [45].

In the two larger studies, the PRORATA [42] and the PASS [47], the 60-day mortality also was provided. In the PRORATA, the 60-day mortality was 3.8 % higher in the PCT-guided group, which represents a 10 % increase in the relative risk of death. However, both the design and the power cannot exclude a negative impact on mortality attributable to the PCT strategy [50]. In the PASS study, the 60-day mortality was similar: 38.2 % vs. 36.9 %.

#### Associated costs

It is well known that a prolonged duration of antibiotic therapy is associated with increased costs. Conversely, the measurement of PCT also is very expensive. The analysis of the cost-benefit of the implementation of a PCT algorithm was not performed in any of the selected studies.

## Conclusions

Biomarkers of infection, namely CRP and PCT, are potentially very useful in the diagnosis of infection as well as in the assessment of its response to antibiotic therapy. Presently, no RCTs of CRP-guided therapy in critically ill patients have been performed. Regarding PCT, in several RCTs, PCT guidance was repeatedly associated with a decrease in the duration of antibiotic therapy. However, these trials present several limitations, namely high rate of patients’ exclusion, high rate of algorithm overruling, long duration of antibiotic therapy in the control group, disregard the effect of renal failure or use of RRT on PCT levels, and above all a possible higher mortality and late organ failure in the PCT arm. In addition, some infections as well as frequent nosocomial bacteria were not evaluated. As a result, in critically ill patients, we cannot recommend the routine use of PCT-guided algorithms of antibiotic stewardship to assist the clinical decision-making process at the bedside. However, biomarkers, namely PCT and CRP but also platelet count, d-dimer, and prothrombin time, could be very useful at the bedside but should never be used solely. Biomarkers should always be used in conjunction with a complete clinical, laboratory, and radiologic evaluation and with a perfect knowledge of its biology, strengths, and limitations. Future studies in this area of knowledge are needed, but their design should take into account the issues identified in the present review to define clearly the role of biomarkers at the beside.

## Abbreviations

CRP: C-reactive protein; ICU: Intensive care unit; LOS: Length of stay; PCT: Procalcitonin; RCT: Randomized controlled trial; RRT: Renal replacement therapy; VAP: Ventilator associated pneumonia.

## Competing interests

PP has received honoraria and has served as advisor of Astra Zeneca, Gilead, Janssen-Cilag, Merck Sharp & Dohme, Novartis, and Pfizer and has received unrestricted grants from Brahms and Virogates. JIFS declares that he has no competing interests.

## Authors’ contributions

PP and JIFS contributed to the study conception and design, performed and participated in the acquisition and interpretation of data, and drafted the manuscript. All authors read and approved the final manuscript.

## Authors’ information

PP is coordinator of the Polyvalent Intensive Care Unit, São Francisco Xavier Hospital, and Professor of Medicine, Faculty of Medical Sciences, New University of Lisbon, Lisbon, Portugal. JIFS is medical researcher from the D’Or Institute for Research and Education, and coordinator of the Postgraduation Program, Instituto Nacional de Câncer, Rio de Janeiro, Brazil.

## References

[B1] Angus DC Linde-Zwirble WT Lidicker J Clermont G Carcillo J Pinsky MR Epidemiology of severe sepsis in the United States: analysis of incidence, outcome, and associated costs of care Crit Care Med 2001 29 1303 1310 10.1097/00003246-200107000-00002 11445675

[B2] Martin GS Mannino DM Eaton S Moss M The epidemiology of sepsis in the United States from 1979 through 2000 N Engl J Med 2003 348 1546 1554 10.1056/NEJMoa022139 12700374

[B3] Agency for Healthcare Research and Quality Diagnostic groups with rapidly increasing costs by payer. 2001–2007. HCUP statistical brief 91 http://www.hcup-us.ahrq.gov/reports/statbriefs/sb91.pdf 21413208

[B4] The outcome of patients with sepsis and septic shock presenting to emergency departments in Australia and New Zealand Crit Care Resusc 2007 9 8 18 17352661

[B5] Povoa PR Carneiro AH Ribeiro OS Pereira AC Influence of vasopressor agent in septic shock mortality. Results from the Portuguese Community-Acquired Sepsis Study (SACiUCI study) Crit Care Med 2009 37 410 416 10.1097/CCM.0b013e3181958b1c 19114885

[B6] Levy MM Dellinger RP Townsend SR Linde-Zwirble WT Marshall JC Bion J Schorr C Artigas A Ramsay G Beale R Parker MM Gerlach H Reinhart K Silva E Harvey M Regan S Angus DC The Surviving Sepsis Campaign: results of an international guideline-based performance improvement program targeting severe sepsis Intensive Care Med 2010 36 222 231 10.1007/s00134-009-1738-3 20069275 PMC2826633

[B7] Ferrer R Artigas A Suarez D Palencia E Levy MM Arenzana A Perez XL Sirvent JM Effectiveness of treatments for severe sepsis: a prospective, multicenter, observational study Am J Respir Crit Care Med 2009 180 861 866 10.1164/rccm.200812-1912OC 19696442

[B8] Singh N Rogers P Atwood CW Wagener MM Yu VL Short-course empiric antibiotic therapy for patients with pulmonary infiltrates in the intensive care unit. A proposed solution for indiscriminate antibiotic prescription Am J Respir Crit Care Med 2000 162 505 511 10934078 10.1164/ajrccm.162.2.9909095

[B9] Weiss CH Moazed F McEvoy CA Singer BD Szleifer I Amaral LA Kwasny M Watts CM Persell SD Baker DW Sznajder JI Wunderink RG Prompting physicians to address a daily checklist and process of care and clinical outcomes: a single-site study Am J Respir Crit Care Med 2011 184 680 686 10.1164/rccm.201101-0037OC 21616996 PMC3208596

[B10] Christ-Crain M Opal SM Clinical review: the role of biomarkers in the diagnosis and management of community-acquired pneumonia Crit Care 2010 14 203 10.1186/cc8155 20236471 PMC2875480

[B11] Pierrakos C Vincent JL Sepsis biomarkers: a review Crit Care 2010 14 R15 10.1186/cc8872 20144219 PMC2875530

[B12] Kumar A Roberts D Wood KE Light B Parrillo JE Sharma S Suppes R Feinstein D Zanotti S Taiberg L Gurka D Cheang M Duration of hypotension before initiation of effective antimicrobial therapy is the critical determinant of survival in human septic shock Crit Care Med 2006 34 1589 1596 10.1097/01.CCM.0000217961.75225.E9 16625125

[B13] Povoa P Serum markers in community-acquired pneumonia and ventilator-associated pneumonia Curr Opin Infect Dis 2008 21 157 162 10.1097/QCO.0b013e3282f47c32 18317039

[B14] Marshall JC Vincent JL Fink MP Cook DJ Rubenfeld G Foster D Fisher CJ Faist E Reinhart K Measures, markers, and mediators: toward a staging system for clinical sepsis. A report of the Fifth Toronto Sepsis Roundtable, Toronto, Ontario, Canada, October 25–26, 2000 Crit Care Med 2003 31 1560 1567 10.1097/01.CCM.0000065186.67848.3A 12771633

[B15] Chastre J Wolff M Fagon JY Chevret S Thomas F Wermert D Clementi E Gonzalez J Jusserand D Asfar P Perrin D Fieux F Aubas S Comparison of 8 vs 15 days of antibiotic therapy for ventilator-associated pneumonia in adults: a randomized trial JAMA 2003 290 2588 2598 10.1001/jama.290.19.2588 14625336

[B16] Micek ST Ward S Fraser VJ Kollef MH A randomized controlled trial of an antibiotic discontinuation policy for clinically suspected ventilator-associated pneumonia Chest 2004 125 1791 1799 10.1378/chest.125.5.1791 15136392

[B17] Pepys MB Hirschfield GM C-reactive protein: a critical update J Clin Invest 2003 111 1805 1812 12813013 10.1172/JCI18921 PMC161431

[B18] Gabay C Kushner I Acute-phase proteins and other systemic responses to inflammation N Engl J Med 1999 340 448 454 10.1056/NEJM199902113400607 9971870

[B19] Hogarth MB Gallimore R Savage P Palmer AJ Starr JM Bulpitt CJ Pepys MB Acute phase proteins, C-reactive protein and serum amyloid A protein, as prognostic markers in the elderly inpatient Age Ageing 1997 26 153 158 10.1093/ageing/26.2.153 9177673

[B20] Jaye DL Waites KB Clinical applications of C-reactive protein in pediatrics Pediatr Infect Dis J 1997 16 735 746 10.1097/00006454-199708000-00003 9271034

[B21] Povoa P C-reactive protein: a valuable marker of sepsis Intensive Care Med 2002 28 235 243 10.1007/s00134-002-1209-6 11904651

[B22] Vigushin DM Pepys MB Hawkins PN Metabolic and scintigraphic studies of radioiodinated human C-reactive protein in health and disease J Clin Invest 1993 91 1351 1357 10.1172/JCI116336 8473487 PMC288106

[B23] Dahaba AA Rehak PH List WF Procalcitonin and C-reactive protein plasma concentrations in nonseptic uremic patients undergoing hemodialysis Intensive Care Med 2003 29 579 583 12652350 10.1007/s00134-003-1664-8

[B24] Salluh JI Soares M Coelho LM Bozza FA Verdeal JC Castro-Faria-Neto HC eSilva JR Bozza PT Povoa P Impact of systemic corticosteroids on the clinical course and outcomes of patients with severe community-acquired pneumonia: a cohort study J Crit Care 2011 26 193 200 10.1016/j.jcrc.2010.07.014 20889284

[B25] Povoa P Souza-Dantas VC Soares M Salluh JF C-reactive protein in critically ill cancer patients with sepsis: influence of neutropenia Crit Care 2011 15 R129 10.1186/cc10242 21595932 PMC3218995

[B26] Reinhart K Karzai W Meisner M Procalcitonin as a marker of the systemic inflammatory response to infection Intensive Care Med 2000 26 1193 1200 10.1007/s001340000624 11089742 PMC7095266

[B27] Oberhoffer M Vogelsang H Jager L Reinhart K Katacalcin and calcitonin immunoreactivity in different types of leukocytes indicate intracellular procalcitonin content J Crit Care 1999 14 29 33 10.1016/S0883-9441(99)90005-9 10102721

[B28] Dandona P Nix D Wilson MF Aljada A Love J Assicot M Bohuon C Procalcitonin increase after endotoxin injection in normal subjects J Clin Endocrinol Metab 1994 79 1605 1608 10.1210/jc.79.6.1605 7989463

[B29] Brunkhorst FM Heinz U Forycki ZF Kinetics of procalcitonin in iatrogenic sepsis Intensive Care Med 1998 24 888 889 10.1007/s001340050683 9757936

[B30] Schuttrumpf S Binder L Hagemann T Berkovic D Trumper L Binder C Utility of procalcitonin concentration in the evaluation of patients with malignant diseases and elevated C-reactive protein plasma concentrations Clin Infect Dis 2006 43 468 473 10.1086/505394 16838236

[B31] Amour J Birenbaum A Langeron O Le Manach Y Bertrand M Coriat P Riou B Bernard M Hausfater P Influence of renal dysfunction on the accuracy of procalcitonin for the diagnosis of postoperative infection after vascular surgery Crit Care Med 2008 36 1147 1154 10.1097/CCM.0b013e3181692966 18379240

[B32] Mongardon N Lemiale V Perbet S Dumas F Legriel S Guerin S Charpentier J Chiche JD Mira JP Cariou A Value of procalcitonin for diagnosis of early onset pneumonia in hypothermia-treated cardiac arrest patients Intensive Care Med 2010 36 92 99 10.1007/s00134-009-1681-3 19844695

[B33] Dahaba AA Elawady GA Rehak PH List WF Procalcitonin and proinflammatory cytokine clearance during continuous venovenous haemofiltration in septic patients Anaesth Intensive Care 2002 30 269 274 12075632 10.1177/0310057X0203000302

[B34] Evaluation of Biomarkers and Surrogate Endpoints in Chronic Disease 2010 The National Academies Press, Washington 25032382

[B35] Gill CW Bush WS Burleigh WM Fischer CL An evaluation of a C-reactive protein assay using a rate immunonephelometric procedure Am J Clin Pathol 1981 75 50 55 6779624 10.1093/ajcp/75.1.50

[B36] Enguix A Rey C Concha A Medina A Coto D Dieguez MA Comparison of procalcitonin with C-reactive protein and serum amyloid for the early diagnosis of bacterial sepsis in critically ill neonates and children Intensive Care Med 2001 27 211 215 10.1007/s001340000709 11280637

[B37] Snider RH Nylen ES Becker KL Procalcitonin and its component peptides in systemic inflammation: immunochemical characterization J Investig Med 1997 45 552 560 9444882

[B38] Christ-Crain M Muller B Procalcitonin in bacterial infections–hype, hope, more or less? Swiss Med Wkly 2005 135 451 460 16208582 10.4414/smw.2005.11169

[B39] Christ-Crain M Muller B Biomarkers in respiratory tract infections: diagnostic guides to antibiotic prescription, prognostic markers and mediators Eur Respir J 2007 30 556 573 10.1183/09031936.00166106 17766633

[B40] Schuetz P Albrich W Christ-Crain M Chastre J Mueller B Procalcitonin for guidance of antibiotic therapy Expert Rev Anti Infect Ther 2010 8 575 587 10.1586/eri.10.25 20455686

[B41] Nobre V Harbarth S Graf JD Rohner P Pugin J Use of procalcitonin to shorten antibiotic treatment duration in septic patients: a randomized trial Am J Respir Crit Care Med 2008 177 498 505 18096708 10.1164/rccm.200708-1238OC

[B42] Bouadma L Luyt CE Tubach F Cracco C Alvarez A Schwebel C Schortgen F Lasocki S Veber B Dehoux M Bernard M Pasquet B Regnier B Brun-Buisson C Chastre J Wolff M Use of procalcitonin to reduce patients' exposure to antibiotics in intensive care units (PRORATA trial): a multicentre randomised controlled trial Lancet 2010 375 463 474 10.1016/S0140-6736(09)61879-1 20097417

[B43] Svoboda P Kantorova I Scheer P Radvanova J Radvan M Can procalcitonin help us in timing of re-intervention in septic patients after multiple trauma or major surgery? Hepatogastroenterology 2007 54 359 363 17523274

[B44] Schroeder S Hochreiter M Koehler T Schweiger AM Bein B Keck FS von Spiegel T Procalcitonin (PCT)-guided algorithm reduces length of antibiotic treatment in surgical intensive care patients with severe sepsis: results of a prospective randomized study Langenbecks Arch Surg 2009 394 221 226 10.1007/s00423-008-0432-1 19034493

[B45] Stolz D Smyrnios N Eggimann P Pargger H Thakkar N Siegemund M Marsch S Azzola A Rakic J Mueller B Tamm M Procalcitonin for reduced antibiotic exposure in ventilator-associated pneumonia: a randomised study Eur Respir J 2009 34 1364 1375 10.1183/09031936.00053209 19797133

[B46] Hochreiter M Kohler T Schweiger AM Keck FS Bein B von Spiegel T Schroeder S Procalcitonin to guide duration of antibiotic therapy in intensive care patients: a randomized prospective controlled trial Crit Care 2009 13 R83 10.1186/cc7903 19493352 PMC2717450

[B47] Jensen JU Hein L Lundgren B Bestle MH Mohr TT Andersen MH Thornberg KJ Loken J Steensen M Fox Z Tousi H Soe-Jensen P Lauritsen AO Strange D Petersen PL Reiter N Hestad S Thormar K Fjeldborg P Larsen KM Drenck NE Ostergaard C Kjaer J Grarup J Lundgren JD Procalcitonin-guided interventions against infections to increase early appropriate antibiotics and improve survival in the intensive care unit: a randomized trial Crit Care Med 2011 39 2048 2058 10.1097/CCM.0b013e31821e8791 21572328

[B48] Miller FG Silverman HJ The ethical relevance of the standard of care in the design of clinical trials Am J Respir Crit Care Med 2004 169 562 564 10.1164/rccm.200311-1577CP 14701713

[B49] Jensen JU Heslet L Jensen TH Espersen K Steffensen P Tvede M Procalcitonin increase in early identification of critically ill patients at high risk of mortality Crit Care Med 2006 34 2596 2602 10.1097/01.CCM.0000239116.01855.61 16915118

[B50] Gibot S Procalcitonin in intensive care units: the PRORATA trial Lancet 2010 375 1605 1606 author reply 1606–1607 20452514 10.1016/S0140-6736(10)60696-4

